# Primary Mental Health Care in a New Era

**DOI:** 10.3390/healthcare10102025

**Published:** 2022-10-14

**Authors:** Athanasios Tselebis, Argyro Pachi

**Affiliations:** Psychiatric Department, “Sotiria” General Hospital of Chest Diseases, 11527 Athens, Greece

Clinical experience and scientific studies highlight the pivotal role that primary health care services have and should have as a gateway to the health care system and as a first point of contact for patients with mental disorders, particularly—but not exclusively—for patients with a disorder in the spectrum of common mental disorders [1,2]. The role of primary mental health care is early diagnosis and intervention, assessment, treatment planning, referral, monitoring, and prevention at the individual and community level [3]. The principles underlying primary mental health care are accessibility; integrated, coordinated, and continuous care with efficiency; reduction in disparities and improvement in well-being for all; and respect for human rights [4]. A variety of cost-effective mental health delivery models for primary care are available, such as collaborative care [5] and various integrated care models [6].

The population’s mental health has deteriorated during the COVID-19 pandemic with the high prevalence of psychological distress forewarning increased rates of mental health disorders [7]. Additionally, the pandemic inadvertently induced global economic recession while at the same time widening pre-pandemic health, social, and economic inequalities [8]. As a consequence, vulnerable populations have been disproportionately afflicted in the COVID-19 era [9]. People suffering from a mental health disorder or disability [10,11], those who are educationally or economically disadvantaged, racial and ethnic minorities, refugees, migrants [12], the homeless, minors, and the elderly are among those adversely affected. Additionally, nontraditional cases are seeking psychosocial counselling, including employed parents as well as school and university students. The effects of the pandemic on diverse populations require the implementation of targeted approaches to mental health care delivery. Scheduling studies without delay by following a pragmatic approach to research, considering current views and experiences and concentrating on providing insightful and practical information in order to prioritize mental health needs among vulnerable populations and effectively allocate resources, is imperative.

Primary mental health care providers are dealing first and foremost with the mental health consequences induced by the pandemic [13]. Managing increasing demand in general practice, primary care professionals have promptly applied digital mental health services to expand the reach and accessibility of mental health care. Digital interventions, such as online platforms, are extensively being used to provide help and support to people with mental health needs [14]. Teleconsultations via telephone or videoconference replaced, as an effective alternative, in-person medical counseling, providing cost-effective interventions remotely while providing immediate access to health care [15,16]. During the changing context of the pandemic, alternative methods of communication were adopted to approach individuals who did not actively access medical care. Screening for and contacting susceptible individuals, organizing and engaging government-provided community services, and monitoring enlisted populations to ensure equitable access to mental health care are of outmost importance. In the planning and organization of health care, involving health care professionals from physicians to community health workers required strengthening personnel, enhancing family practitioners’ mental health training, and expanding public psychological services, including through digital modalities, to alleviate emotional distress and overcome adversities. Concerns were raised for individuals recovering from the SARS-CoV-2 virus and their caregivers [17], health care staff working on the front lines [18], and socially diverse populations markedly affected by varying degrees of lockdown protocols, such as frail geriatric individuals, people dismissed from employment, working mothers, and youth. It remains unknown whether the implemented programs can keep up with the increasing demands arising during the pandemic.

Enduring challenges emerge when attempting to integrate mental health care within the primary health care system in today’s environment. Several issues originated during the pandemic, while others pre-existed and were aggravated [19]. A survey conducted by the World Health Organization disclosed that the vast majority (93%) of 130 countries suspended outpatient and community mental health services [20]. Meanwhile, ministerial recommendations insisted on enhanced cooperation and teamwork between primary and community mental health services with secondary specialized mental health care, emphasizing the need for community services’ contribution in quality health interventions, and underscoring mental health care in primary and community-based settings. Few recent articles have investigated how best to overcome these barriers in the post-COVID-19 era [21]. Addressing emerging barriers and challenges to treating common mental health conditions and interventions to enhance the primary care of mental health disorders, aiming to review how mental health needs might be managed in the post-COVID-19 era, is a priority.

By the time this Special Issue was launched, the world was responding to the COVID-19 pandemic and already confronting significant mental health problems. Other diseases with distinct epidemiological profiles along with socioeconomic factors constitute the background wherein pandemics prevail. The syndemic approach in the field of mental diseases captures the individual experiences and the environmental socioeconomic impacts, influencing the direction of clinical practice, policy development, and research priorities and dictating what types of interventions matter [22]. Evidence suggests that implementing community interventions may mitigate the adverse mental health consequences of these environmental risk factors [23]. Promoting the COVID-19 vaccination program, financial support measures to counteract recession and inflation, addressing stigma issues reinforced during the pandemic, building the national response framework for pandemic conditions including plans for mental health services, and the dissemination of computerized transformation and innovation are among adaptive responses in different countries to cope with the mental health repercussions.

Global pandemics, climate change, war, and socioeconomic and energy crises outline a frightful environmental scenario. Contextual issues and the impact of social determinants should primarily be considered when implementing practical strategies and promoting interventions. Nowadays, more than ever, mental health care delivery needs a paradigm shift, for pragmatic applications at the clinical level and for scientific purposes, promoting biopsychosocial integrated mental health care with an emphasis on community-based psychosocial support [24]. Recognition of the central role of environmental context in mental health outcomes and earlier mental health interventions at the community level, rather than only at the individual level, serves as a prerequisite in order to reform mental health research and service delivery [25]. According to this transformational shift, social and psychological needs can be addressed at the community level so that fewer people access hospital clinics with staff that may be overwhelmed in dealing with the structural and social issues contributing to patients’ mental health needs.

Unfortunately, the mental health care system has always focused on treating the acute phase of psychiatric disorders, minimizing and questioning the effectiveness of mental health prevention and promotion processes. Specific primary prevention approaches (universal, selective, and indicated), or the “stepped care” approach, mostly remain theoretical formulations either fragmentally applied or somehow absent from practical strategies and interventions. The literature suggests that several risk factors are interrelated and tend to have synergistic effects, such as social risk factors combined with the pandemic-induced stressors [26]. People with mental health disorders or disabilities and people who have already been exposed to multiple risk factors may be less able to cope with their impacts, compared to those who have never been exposed to any risk factors. The cumulative effects of environmental risk factors in genetically vulnerable populations increase the likelihood of developing a mental disorder. Data and research are needed to identify the multiple pathways through which risks contribute to a diversity of adverse outcomes among various populations. In these circumstances, the results of epidemiological studies and interventions aimed at understanding the nature of the disease, especially for multifactorial disorders, and detecting or intervening in environmental, social, and other factors involved in the etiopathogenesis of the disorders, are of outmost importance and have practical application.

Since exposure to environmental stressors is unavoidable, strengthening the protective factors that contribute to psychological resilience serves in adaptive coping. Preventive interventions aim to implement strategies, allocate available resources, and modify environmental factors so that they do not cause stress but benefit people’s wellbeing. The determinants that compromise mental health are largely beyond the health sector, making effective initiatives more complex and difficult. This underlines the urgent need for interdisciplinary collaboration, multidisciplinary engagement, and transdisciplinary approaches, in order to develop comprehensive strategies to address needs on a global scale. In addition, mental health promotion and prevention call for governments and policy makers to fully recognize the impact of poverty and social disadvantage on the mental health of the population [27]. Changing our perspective about public mental health care requires skills such as assessing community needs, identifying and prioritizing high-risk groups, and intervening with methods such as counseling, training, and crisis intervention. In this way, by providing people more accessible and effective alternatives in a timely manner, we can prevent emergency mental health issues needing urgent secondary mental health care. Addressing persistent and systemic gaps in the mental health delivery system [28] demands reform and structural changes at the institutional, organizational, and administrative level and a package of feasible, safe, and cost-effective community-based interventions with short- and long-term benefits beyond mental health outcomes (educational, functional, and societal).

## References

[1] (2008). What is primary care mental health?: WHO and Wonca Working Party on Mental Health. Ment. Health Fam. Med..

[2] Kyanko K.A., ACurry L., EKeene D., Sutherland R., Naik K., Busch S.H. (2022). Does Primary Care Fill the Gap in Access to Specialty Mental Health Care? A Mixed Methods Study. J. Gen. Intern. Med..

[3] Ashcroft R., Donnelly C., Dancey M., Gill S., Lam S., Kourgiantakis T., Adamson K., Verrilli D., Dolovich L., Kirvan A. (2021). Primary care teams’ experiences of delivering mental health care during the COVID-19 pandemic: A qualitative study. BMC Fam. Pract..

[4] Gómez-Restrepo C., Cepeda M., Torrey W.C., Suarez-Obando F., Uribe-Restrepo J.M., Park S., Acosta M.P.J., Camblor P.M., Castro S.M., Aguilera-Cruz J. (2022). Perceived access to general and mental healthcare in primary care in Colombia during COVID-19: A cross-sectional study. Front. Public Health.

[5] Moise N., Wainberg M., Shah R.N. (2021). Primary care and mental health: Where do we go from here?. World J. Psychiatry.

[6] Ulupinar D. (2021). The need for integrated primary and behavioral healthcare care in the post-pandemic era. Asian J. Psychiatry.

[7] Chennapragada L., Sullivan S.R., Hamerling-Potts K.K., Tran H., Szeszko J., Wrobleski J., Mitchell E.L., Walsh S., Goodman M. (2022). International PRISMA scoping review to understand mental health interventions for depression in COVID-19 patients. Psychiatry Res..

[8] Gong Y., Liu X., Zheng Y., Mei H., Que J., Yuan K., Yan W., Shi L., Meng S., Bao Y. (2022). COVID-19 Induced Economic Slowdown and Mental Health Issues. Front. Psychol..

[9] Nam S.H., Nam J.H., Kwon C.Y. (2021). Comparison of the Mental Health Impact of COVID-19 on Vulnerable and Non-Vulnerable Groups: A Systematic Review and Meta-Analysis of Observational Studies. Int. J. Environ. Res. Public Health.

[10] Bahji A., Bach P., Danilewitz M., El-Guebaly N., Doty B., Thompson L., Clarke D.E., Ghosh S.M., Crockford D. (2021). Strategies to aid self-isolation and quarantine for individuals with severe and persistent mental illness during the COVID-19 pandemic: A systematic review. Psychiatr. Res. Clin. Pract..

[11] Pachi A., Tselebis A., Ilias I., Tsomaka E., Papageorgiou S.M., Baras S., Kavouria E., Giotakis K. (2022). Aggression, Alexithymia and Sense of Coherence in a Sample of Schizophrenic Outpatients. Healthcare.

[12] Mengesha Z., Alloun E., Weber D., Smith M., Harris P. (2022). “Lived the Pandemic Twice”: A Scoping Review of the Unequal Impact of the COVID-19 Pandemic on Asylum Seekers and Undocumented Migrants. Int. J. Environ. Res. Public Health.

[13] Rohilla J., Tak P., Jhanwar S., Hasan S. (2020). Primary care physician’s approach for mental health impact of COVID-19. J. Fam. Med. Prim. Care.

[14] Rauschenberg C., Schick A., Hirjak D., Seidler A., Paetzold I., Apfelbacher C., Riedel-Heller S.G., Reininghaus U. (2021). Evidence Synthesis of Digital Interventions to Mitigate the Negative Impact of the COVID-19 Pandemic on Public Mental Health: Rapid Meta-review. J. Med. Internet Res..

[15] Chen J.A., Chung W.J., Young S.K., Tuttle M.C., Collins M.B., Darghouth S.L., Longley R., Levy R., Razafsha M., Kerner J.C. (2020). COVID-19 and telepsychiatry: Early outpatient experiences and implications for the future. Gen. Hosp. Psychiatry.

[16] Althumairi A., AlHabib A.F., Alumran A., Alakrawi Z. (2022). Healthcare Providers’ Satisfaction with Implementation of Telemedicine in Ambulatory Care during COVID-19. Healthcare.

[17] Sosa J.V.C., Gonzales F.M.M., Ramirez Z.E.A., Castro M.C., Huancahuire-Vega S. (2022). Depression Associated with Caregiver Quality of Life in Post-COVID-19 Patients in Two Regions of Peru. Healthcare.

[18] Pachi A., Sikaras C., Ilias I., Panagiotou A., Zyga S., Tsironi M., Baras S., Tsitrouli L.A., Tselebis A. (2022). Burnout, Depression and Sense of Coherence in Nurses during the Pandemic Crisis. Healthcare.

[19] Spagnolo J., Beauséjour M., Fleury M.J., Clément J.F., Gamache C., Sauvé C., Couture L., Fleet R., Knight S., Gilbert C. (2022). Perceptions on barriers, facilitators, and recommendations related to mental health service delivery during the COVID-19 pandemic in Quebec, Canada: A qualitative descriptive study. BMC Prim. Care.

[20] Henderson E. (2020). WHO Survey: COVID-19 Halted Mental Health Services in 93% of Countries. https://www.news-medical.net/news/20201005/WHO-survey-COVID-19-halted-mental-health-services-in-9325-of-countries.aspx.

[21] Keyes B., McCombe G., Broughan J., Frawley T., Guerandel A., Gulati G., Kelly B.D., Osborne B., O’Connor K., Cullen W. (2022). Enhancing GP care of mental health disorders post-COVID-19: A scoping review of interventions and outcomes. Ir. J. Psychol. Med..

[22] Di Ciaula A., Krawczyk M., Filipiak K.J., Geier A., Bonfrate L., Portincasa P. (2021). Noncommunicable diseases, climate change and iniquities: What COVID-19 has taught us about syndemic. Eur. J. Clin. Investig..

[23] McGrath M., Duncan F., Dotsikas K., Baskin C., Crosby L., Gnani S., Hunter R.M., Kaner E., Kirkbride J.B., Lafortune L. (2021). School for Public Health Research Public Mental Health Programme. Effectiveness of community interventions for protecting and promoting the mental health of working-age adults experiencing financial uncertainty: A systematic review. J. Epidemiol. Community Health..

[24] Smit D., Hill L., Walton I., Kendall S., De Lepeleire J. (2020). European Forum for Primary Care: Position Paper for Primary Care Mental Health: Time for change, now more than ever!. Prim. Health Care Res. Dev..

[25] Alegría M., Zhen-Duan J., O’Malley I.S., DiMarzio K. (2022). A New Agenda for Optimizing Investments in Community Mental Health and Reducing Disparities. Am. J. Psychiatry.

[26] Boden M., Zimmerman L., Azevedo K.J., Ruzek J.I., Gala S., Abdel Magid H.S., Cohen N., Walser R., Mahtani N.D., Hoggatt K.J. (2021). Addressing the mental health impact of COVID-19 through population health. Clin. Psychol. Rev..

[27] Compton M.T., Shim R.S. (2020). Mental Illness Prevention and Mental Health Promotion: When, Who, and How. Psychiatr. Serv..

[28] Saif-Ur-Rahman K.M., Mamun R., Anwar I. (2019). Identifying gaps in primary healthcare policy and governance in low-income and middle-income countries: Protocol for an evidence gap map. BMJ Open.

